# Plausibility of potassium ion-exchanged ZSM-5 as soot combustion catalysts

**DOI:** 10.1038/s41598-017-03504-3

**Published:** 2017-06-12

**Authors:** Chenxi Lu, Taizheng Liu, Qiaolan Shi, Qian Li, Ying Xin, Lei Zheng, Zhaoliang Zhang

**Affiliations:** 1 School of Chemistry and Chemical Engineering, Shandong Provincial Key Laboratory of Fluorine Chsemistry and Chemical Materials, University of Jinan, No. 336, West Road of Nan Xinzhuang, Jinan, 250022 China; 2 Institute of High Energy Physics, Chinese Academy of Sciences, Beijing, 100049 China

## Abstract

Potassium (K) ion-exchanged ZSM-5 zeolites were investigated for catalytic soot combustion. X-ray absorption fine-structure (XAFS), Raman, *in situ* IR and NH_3_-temperature programmed desorption (NH_3_-TPD) confirmed the location of K^+^ at the ion-exchanged sites. Temperature-programmed oxidation (TPO) reactions showed that K-ZSM-5 decreased ignition tempeatures of soot combustion and increased selectivity to CO_2_. The improved activity for soot combustion by increasing K^+^-exchanged amounts via decreasing the Si/Al ratio reinforced the K^+^ ions participating in soot combustion. ^18^O_2_ isotopic isothermal reactions suggested the activation of gaseous oxygen by the K^+^ ions. This demonstrated a new appliction of alkali metal exchanged zeolites and the strategy for enhancement of catalytic soot combustion activity.

## Introduction

Soot particulates are one of the main pollutants emitted from diesel engines, which represents a significant threat to environment and human health. For instance, soot can not only cause climate changes but also be easily deposited on lungs increasing cancer risk^1, 2^. Currently, diesel particulate filters (DPF) are considered to be the most efficient way to eliminate soot from diesel engine exhaust^3–5^. One of the great challenges for DPF is to find a robust catalyst to decrease ignition temperatures of the deposited soot. Up to now, many kinds of catalysts have been investigated and employed to catalyze soot combustion. Among them, noble metal-based catalysts have been widely used due to their excellent catalytic activity at low temperatures^6–8^. For instance, Zhao’s group has reported that gold nanoparticles supported on three-dimensionally ordered macroporous oxides exhibit outstanding activity^9^. However, much work has been focused on the oxide catalysts^10–13^. ZSM-5 as a type of zeolite oxides with a well-defined three-dimensional micropore structure and a capability for cation exchange, has already become an applied catalyst for a variety of chemical processes^14–17^. Recently, Pt/H-ZSM-5 was reported for soot combustion by Liu *et al*.^18, 19^, who found that the acidic ZSM-5 support inhibited NO_2_ adsorption. However, owing to the high price of noble metals, K-promoted oxide catalysts have attracted much attention^20–23^. Specially, Kimura *et al*. reported that K_2_CO_3_ supported on aluminosilicate zeolite exhibited excellent catalytic activity^24, 25^. Until now, no one has reported ion-exchanged K-ZSM-5 as catalysts for soot combustion.

Here, K-ZSM-5 was first reported to increase both activity and selectivity to CO_2_ for soot combustion. This was confirmed by the fact that increasing K^+^-exchanged amounts via decreasing the Si/Al ratio led to the improved activity, which was attributed to the activation of gaseous oxygen by K^+^ at the ion-exchanged sites.

## Results

### Characterizations

**Figure 1:**
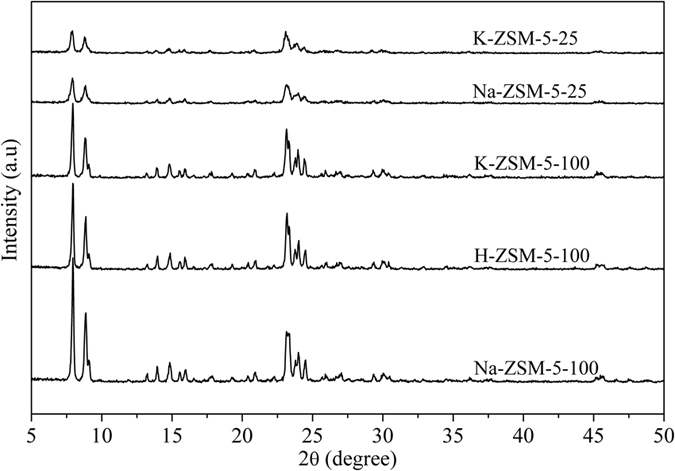
XRD patterns of the samples.

Na-ZSM-5 zeolites were hydrothermally synthesized with Si/Al ratios of 100 (Na-ZSM-5-100) and 25 (Na-ZSM-5-25)^16^, followed by ion-exchanges of H^+^ and K^+^, which results in H-ZSM-5-100 and K-ZSM-5-100 (25) samples, respectively. Figure 1 shows the powder X-ray powder diffraction (XRD) patterns of all samples. Na-ZSM-5-100 and Na-ZSM-5-25 exhibited the typical diffraction peaks of MFI zeolite structure, confirming the formation of a crystalline ZSM-5-type zeolite. After ion-exchange with H^+^ and K^+^ cations, all samples remained the MFI structure. Notably, no crystal phases related to K species were detected, indicating that the K^+^ ions are highly dispersed in zeolites, the same as that in literature^16, 26^. The zeolite crystallite sizes estimated using Scherrer’s equation are between 45 and 56 nm (Table 1).

**Table 1:** Physicochemical properties of the samples.

Sample	BET area (m^2^/g)^a^	Si molar percent (%)^b^	Al molar percent (%)^b^	K molar percent (%)^b^	Primary particle size (nm)^c^
H-ZSM-5-100	365.0	32.87	0.31	–	56
K-ZSM-5-100	346.5	32.47	0.30	0.260	58
K-ZSM-5-25	405.2	12.18	0.48	0.420	45

^a^Determined from the N_2_ adsorption/desorption isotherms; ^b^Determined from the ICP–AES analysis; ^c^Calculated using Scherrer equation.

**Figure 2:**
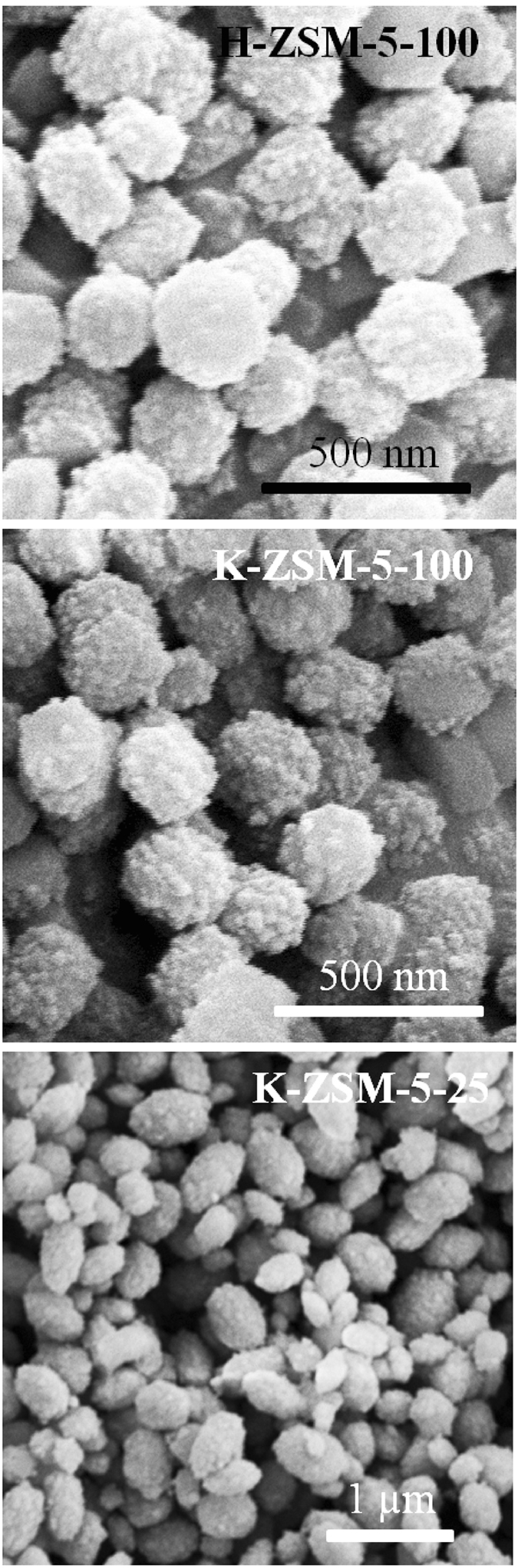
SEM images of the samples.

Figure 2 shows scanning electron microscopy (SEM) images of the typical samples. All of them exhibited 200–400 nm spheres, which consist of little primary particles as detected by XRD (Table 1). Furthermore, no significant morphological changes were observed after ion exchange. All samples demonstrated high surface areas (Table 1), which is expected for a ZSM-5-type zeolite. N_2_ adsorption/desorption isotherms and the pore size distribution displayed microporous structure predominantly (Supplementary Figure S1). Since soot particles often possess a big size (larger than 25 nm), it was concluded that the solid soot can hardly diffuse into the inner pores of the zeolite^27, 28^.

Inductively coupled plasma-atomic emission spectrometer (ICP–AES) results are shown in Table 1. K-ZSM-5-100 has a low K content because of the high Si to Al atomic ratio. The substitution of Si^4+^ by Al^3+^ in the SiO_2_ frameworks generates the negative charges on the oxygen atoms of the framework, which needs the positive charge to balance. The alkali metal cations exist in ZSM-5 in order to compensate the charge imbalance^29, 30^. For K-ZSM-5-100, the K content is near to Al. In order to improve K content, the Si/Al ratio is decreased from 100 to 25. As expected, K-ZSM-5-25 shows a higher K content compared with K-ZSM-5-100.

**Figure 3:**
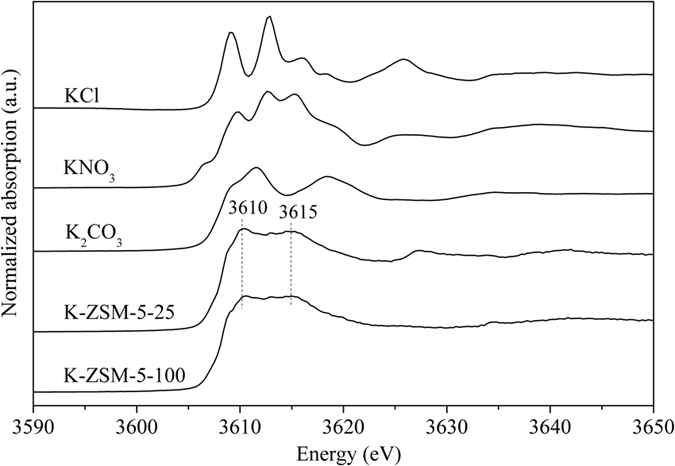
Normalized absorption of K K-edge for K-ZSM-5, K_2_CO_3_, KNO_3_ and KCl.

In order to determine the location of K^+^, IR, Raman and X-ray absorption fine-structure (XAFS) experiments were performed. IR spectra show characteristic bands at 1227, 1109, 804, 555 and 457 cm^−1^ of ZSM-5 (Supplementary Figure S2)^29, 31^. No peaks of surface K species was detected, which is also confirmed by Raman spectroscopy (Supplementary Figure S3). The typical peak corresponding to K_2_CO_3_ at 1063 cm^−1^ is absent on the K-ZSM-5 samples, suggesting that the K^+^ ions are inside zeolite channel, consistent with XRD analysis. Normalized absorption of K K-edge for K-ZSM-5 show two prominent peaks at 3610 eV and 3615 eV (Fig. 3), which is different from those of K_2_CO_3_, KCl and KNO_3_ (for references), but similar to O-K species in glass^32^, suggesting a strong interaction between K^+^ and oxygen and the effect from the coordinated Al and Si in the zeolite. This testified that K^+^ in K-ZSM-5 is located at the ion-exchanged sites.

### Activity

**Figure 4:**
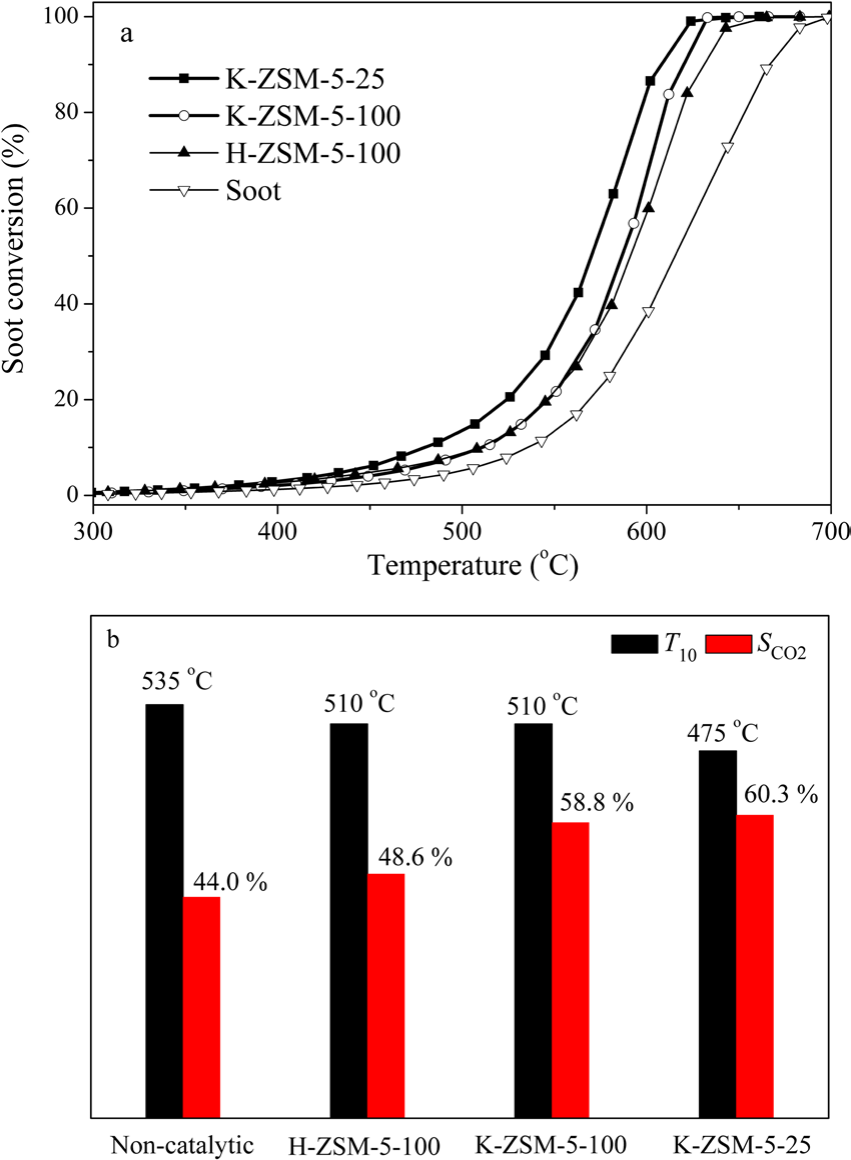
Catalytic performance for soot combustion. (**a**) Soot conversion (%) versus temperature, and (**b**) *T*_10_ and ${S}_{{{\rm{CO}}}_{2}}$ for un-catalyzed, H-ZSM-5-100, K-ZSM-5-100 and K-ZSM-5-25 samples.

The catalytic performance for soot combustion was studied using temperature-programmed oxidation (TPO) in O_2_ atmosphere (Supplementary Figure S4). The soot conversion as a function of temperature over the non-catalytic, H-ZSM-5-100, K-ZSM-5-100 and K-ZSM-5-25 samples were presented in Fig. 4(a). The ignition temperature *T*_10_ (the temperature at which 10% of the soot is converted) and the selectivity to CO_2_ (${S}_{{{\rm{CO}}}_{2}}$) were shown in Fig. 4(b). Non-catalytic soot combustion showed a high *T*_10_ at 535 °C and 44% ${S}_{{{\rm{CO}}}_{2}}$. H-ZSM-5 decreased *T*_10_ to 510 °C with a low ${S}_{{{\rm{CO}}}_{2}}$, suggesting a poor activity for soot combustion. In comparison, *T*_10_ for K-ZSM-5-100 is similar to H-ZSM-5, but the ${S}_{{{\rm{CO}}}_{2}}$ increased to about 60%. As for K-ZSM-5-25, *T*_10_ further decreased to 475 °C in keeping a similarly high ${S}_{{{\rm{CO}}}_{2}}$. Increasing K amount led to a lower ignition temperature, confirming that the activity was improved by ion-exchanged K^+^. After reactions, the structure of all samples remained stable (Supplementary Figure S5).

## Discussion

**Figure 5:**
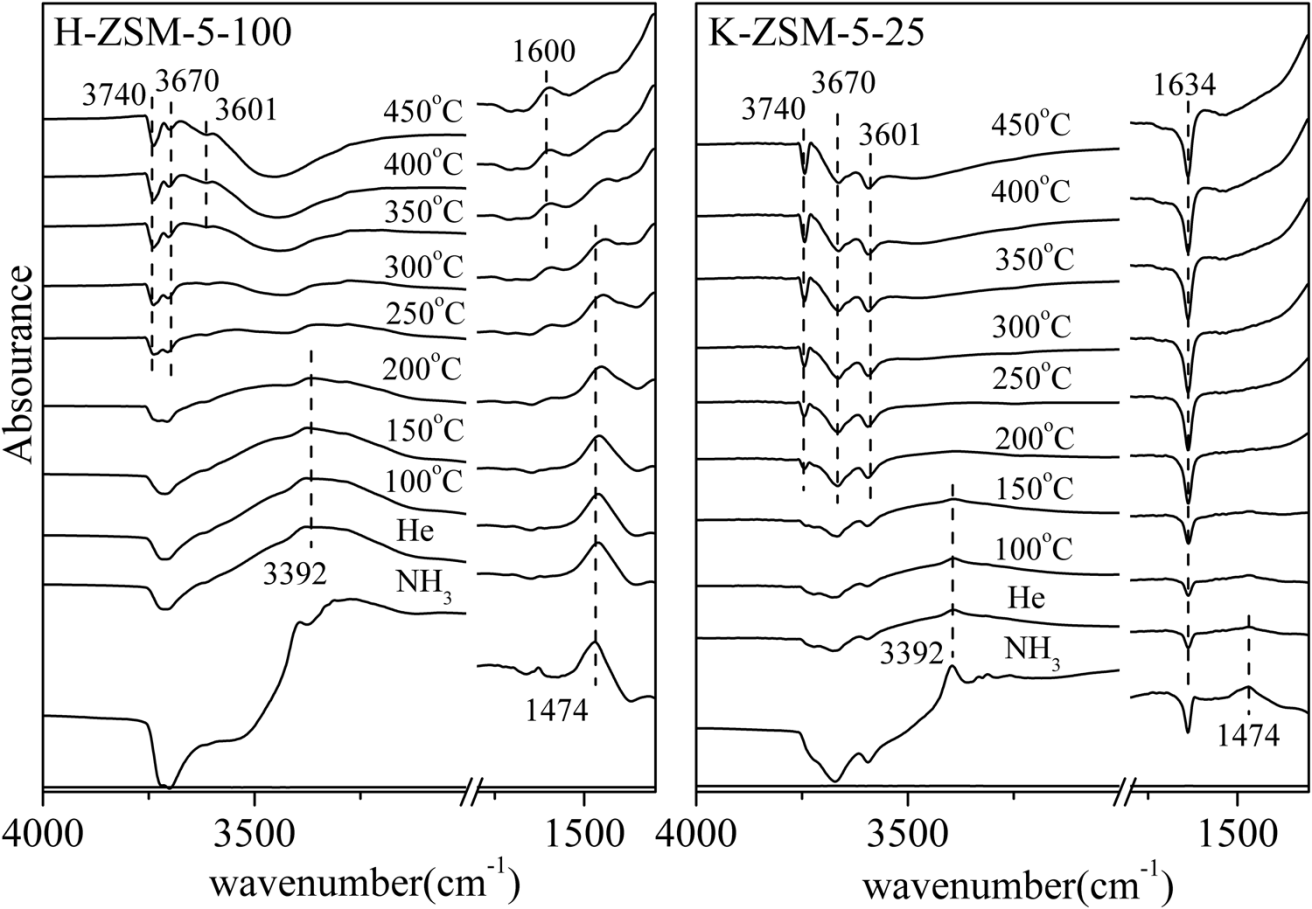
*In situ* IR spectra of NH_3_ desorption in He.

In order to disclose the active nature, *in situ* IR and NH_3_-temperature programmed desorption (NH_3_-TPD) were performed. Figure 5 shows *in situ* IR spectra of NH_3_ desorption with temperature on H-ZSM-5-100 and K^+^-exchanged samples after NH_3_ adsorption. For H-ZSM-5-100, the band at 1474 cm^-1^ was assigned to the bending vibration of NH_4_^+^ on the Brønsted acidic sites^33–35^. The intensity of the band decreased as the temperature increases, corresponding to the presence of 1600 cm^-1^ above 300 °C, which is derived from the desorbed NH_3_ adsorption on Lewis acid sites^33^. The vibration band at 3392 cm^−1^ is attributed to adsorbed NH_3_ on zeolites which disappeared with temperature increasing^36^. In addition, several negative bands at 3740, 3670 and 3601 cm^-1^ were observed. The bands at 3740 cm^−1^ and 3670 cm^−1^ may be assigned to Si-OH and Al-OH vibrations located at the extra framework of zeolites or the external surfaces of microcrystals, respectively, whereas the weak negative band at 3601 cm^−1^ is due to the stretching vibrations of bridging OH group Al–(OH)–Si^34, 37–39^. Compared with H-ZSM-5-100, the negligible 1474 cm^−1^ band for H-ZSM-5-25 disappeared after He purging, suggesting the substitution of H^+^ by K^+^. A new negative band at 1634 cm^−1^ is assigned to the H_2_O bending vibration^40^. All negative bands correspond to NH_3_ adsorbed on the weak Brønsted acidic sites as NH_4_^+^ species.

**Figure 6:**
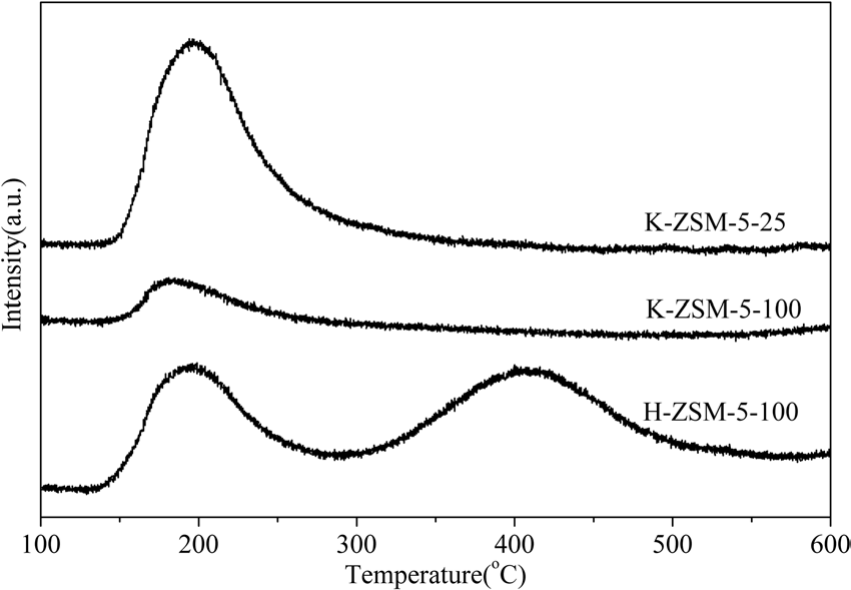
NH_3_-TPD profiles of the samples.

Figure 6 shows NH_3_-TPD profiles of the samples. For H-ZSM-5-100, two NH_3_ desorption peaks were observed at 191 °C (LT) and 415 °C (HT), respectively^41, 42^. LT was assigned to NH_3_ desorption from weak acid sites^43^. According to *in situ* IR results (Fig. 5), HT was attributed to NH_3_ desorption from strong Brønsted acid sites and Lewis acidic sites. In comparison, no HT peak for K-ZSM-5-100 and K-ZSM-5-25 was observed, which confirmed that the original Brønsted acid H^+^ in H-ZSM-5 was substituted by K^+^^44^.

The *T*_10_ of K-ZSM-5-100 is similar to that of H-ZSM-5-100, but the ${S}_{{{\rm{CO}}}_{2}}$ increased, which might be ascirbed to the introduction of K^+^ ions into H-ZSM-5-100. From above characterization results, the Brønsted acid H^+^ sites of H-ZSM-5-100 was substituted by K^+^ leading to the formation of the O-K species. The as-formed O-K species can adsorb and activate gaseous oxygen^45^. In accordance with our previous work, the higher activity and selectivity to CO_2_ were obtained^46^. This indicated that the K^+^ at ion-exchanged sites were active to catalyze soot oxidation. To further reinforce our findings, the amount of exchanged K^+^ was increased by decreasing Si/Al ratio from 100 to 25 and thus K-ZSM-5-25 was prepared. *T*_10_ decreased from 510 °C for K-ZSM-5-100 to 475 °C for K-ZSM-5-25. Meanwhile the ${S}_{{{\rm{CO}}}_{2}}$ kept similarly high. This confirmed that the K^+^ ions at the ion-exchanged sites in ZSM-5 participated in soot combustion.

**Figure 7:**
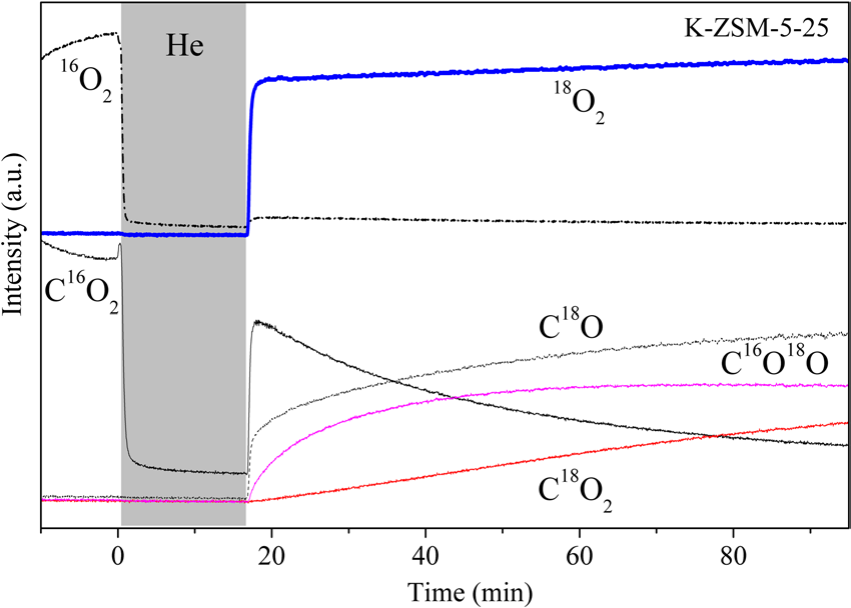
Isothermal reactions for soot combustion at 500 °C after 1%^16^O_2_ was switched to 1% ^18^O_2_ in He on K-ZSM-5-25.

The role of the O-K species to activate gaseous oxygen was investigated by ^18^O_2_ isotopic isothermal reaction at 500 °C. As shown in Fig. 7, before switching form the ^16^O_2_ to ^18^O_2_ (the left of the shadow), the main product was C^16^O_2_, confirming that the soot combustion occurs. Then the sample was purged with He in order to eliminate the residual ^16^O_2_. After switching from the He to ^18^O_2_ (the right of the shadow), the C^16^O_2_ concentration first jumped because K-ZSM-5-25 is prone to adsorb CO_2_^29^, and then decreased rapidly. Comparatively, the products of C^18^O_2_ and C^16^O^18^O increased gradually and reached a stable level. However, non-catalytic soot did not show any response at the same conditions (Supplementary Figure S6). This indicated that the gaseous oxygen has been activated by K-ZSM-5-25. The activation of gaseous oxygen can be attributed to ion-exchanged K^+^ in K-ZSM-5-25 based on above discussion^46^.

## Conclusions

K-ZSM-5 zeolites were prepared by ion-exchange and evaluated for soot combustion. The location of K^+^ at the ion-exchanged sites were confirmed by XAFS, Raman, *in situ* IR and NH_3_-TPD. K-ZSM-5 decreased ignition tempeature of soot combustion and increased selectivity to CO_2_. The improved activity for soot combustion by increasing K^+^-exchanged amount via decreasing the Si/Al ratio reinforced the K^+^ ions participating in soot combustion. The activation of gaseous oxygen by K^+^ ions was testified by ^18^O_2_ isotopic trace.

## Methods

### Sample preparation

Na-ZSM-5 zeolites with Si/Al ratios of 100 (Na-ZSM-5-100) and 25 (Na-ZSM-5-25) were prepared as proposed by Chen *et al*.^16^. As an example, Na-ZSM-5-100 was synthesized with a solution containing 0.0372 g NaAlO_2_, 26.7 mL H_2_O, 11.08 g TPAOH and 10.13 mL TEOS. After stirring for 6 h at room temperature, the resulting solution was transferred into an autoclave 180 °C for 4 days for crystallization. The product was collected and washed by centrifugation, and finally dried at 80 °C. The as-obtained product was further calcined at 550 °C for 5 h in air to remove organic templates. H-ZSM-5-100 and K-ZSM-5-100 samples were prepared from ion-exchanges of NH_4_NO_3_ solution (1 mol/L) and KCl solution (1 mol/L) at 80 °C for 5 h, respectively (In order to decrease Na^+^ concentration in the sample, the ion-exchange process was repeated), followed by centrifugation, washing, drying in air and calcination at 500 °C for 2 h. The K-ZSM-5-25 was prepared in a similar procedure.

### Catalyst characterization

X-ray powder diffraction (XRD) patterns were recorded on a Rigaku D/max-rc diffractometer. Scanning electron microscopy (SEM) images were obtained on a field emission scanning electron microscope (a Hitachi S-2500). Prior to detection, samples were sputtered with a thin layer of gold (Au) with a typical sputtering instrument to improve the surface conductivity. Surface area and pore size distribution were determined by N_2_ adsorption/desorption at 77 K using BET method with a Micromeritics ASAP 2020 instrument after off-gassing at 300 °C for 5 h prior to analysis. Inductively coupled plasma-atomic emission spectrometer (ICP–AES) experiments were carried out on an IRIS Intrepid IIXSP instrument from Thermo Elemental. IR experiments were carried out using a FTIR spectrometer (Bruker Tensor 27) over the range 400–4000 cm^−1^ with 32 scans at a resolution of 4 cm^–1^. The samples were diluted with KBr in a ratio of 1:100. Raman spectroscopy was conducted using a LabRAM HR800 Confocal Raman system with 633 nm diode laser excitation (Raman, LabRAM HR800, HORLBA JY). X-ray absorption fine-structure (XAFS) measurements for the K K-edge were performed on the XAFS station of Beijing synchrotron radiation facility (BSRF, Beijing, China). *In situ* IR spectra were recorded on a Bruker Tensor 27 spectrometer over 1000−4000 cm^−1^ after 32 scans at a resolution of 4 cm^−1^. The sample was pressed into a thin self supporting wafer, which was loaded into an *in situ* infrared transmission cell capable of operating up to 450 °C and equipped with gas flow system. The sample was pretreated at 450 °C for 30 min in He (50 mL/min) and then the background spectrum was recorded in a flowing He atmosphere at 100 °C. NH_3_ was introduced and adsorbed for 30 min. After purging with He, the sample was heated up to 450 °C at a heating rate of 5 °C/min in He (50 mL/min). NH_3_-temperature programmed desorption (NH_3_-TPD) experiments were performed in a quartz reactor using 50 mg catalyst. Prior to experiments, the sample powders in a quartz reactor were pretreated at 500 °C for 30 min under He (50 mL/min) to remove surface impurities and then cooled to 100 °C. The sample was saturated with 4000 ppm of NH_3_/He (50 mL/min) for 30 min and then purged with He. Afterward, the sample was heated up to 600 °C at a heating rate of 10 °C/min under He (50 mL/min). NH_3_ was detected using a quadruple mass spectrometer (MS, OminiStar 200, Balzers). An isotopic isothermal reaction was performed by switching the flowing gas from 1% ^16^O_2_ to 1% ^18^O_2_ diluted in He at 500 °C. Before switching to 1% ^18^O_2_, the sample was purged with He in order to eliminate the residual ^16^O_2_. 50 mg of a mixture of the soot and catalyst (SiO_2_) in a tight contact mode was employed. The effluent gas from the reactor was continuously monitored by a MS.

### Activity measurement

Temperature-programmed oxidation (TPO) reactions were conducted in the fixed bed micro-reactor. Printex–U from Degussa is used as the model soot. The soot was mixed with the catalyst in a weight ratio of 1:9 in an agate mortar for 30 min, which result in a tight contact between soot and catalyst. A 50 mg sample of the soot/catalyst mixture was pretreated in a flow of He (50 mL/min) at 200 °C for 30 min to remove surface-adsorbed species. After cooling down to room temperature, a gas flow with 5 vol.% oxygen in He was introduced and then TPO was started at a heating rate of 5 °C/min until temperature reached at 700 °C. CO and CO_2_ concentrations in the effluent gas were online monitored using a gas chromatograph (GC) (SP-6890, Shandong Lunan Ruihong Chemical Instrument Corporation, China) fitted with a methanator. The ignition temperature for soot combustion is evaluated by the value of *T*_10_, which is defined as the temperature at which 10% of the soot is converted. The selectivity to CO_2_ formation (${S}_{{{\rm{CO}}}_{2}}$) is defined as the percentage CO_2_ outlet concentration divided by the sum of the CO_2_ and CO outlet concentrations.

## Electronic supplementary material

Supplementary Information
