# Supplementary material for: Plausibility of potassium ion-exchanged ZSM-5 as soot combustion catalysts
Source: Sci Rep. 2017 Jun 12;7:3300. doi: 10.1038/s41598-017-03504-3 (PMC5468242; doi:10.1038/s41598-017-03504-3)
Supplement: Supplementary file 1 — Supplementary Information [file 41598_2017_3504_MOESM1_ESM.pdf]

Supporting Information

Plausibility of potassium ion-exchanged ZSM-5 as soot combustion catalysts

Chenxi Lu[1], Taizheng Liu[1], Qiaolan Shi[1], Qian Li[1], Ying Xin[1], Lei Zheng[2], Zhaoliang Zhang[1,*]

[1]*School of Chemistry and Chemical Engineering, Shandong Provincial Key Laboratory of Fluorine Chemistry and Chemical Materials, University of Jinan, No. 336, West Road of Nan Xinzhuang, Jinan 250022, China*

[2]*Institute of High Energy Physics, Chinese Academy of Sciences, Beijing 100049, China*

Corresponding authors: Zhaoliang Zhang

E-mail address: chm_zhangzl@ujn.edu.cn

Tel/Fax: +86 531 89736032

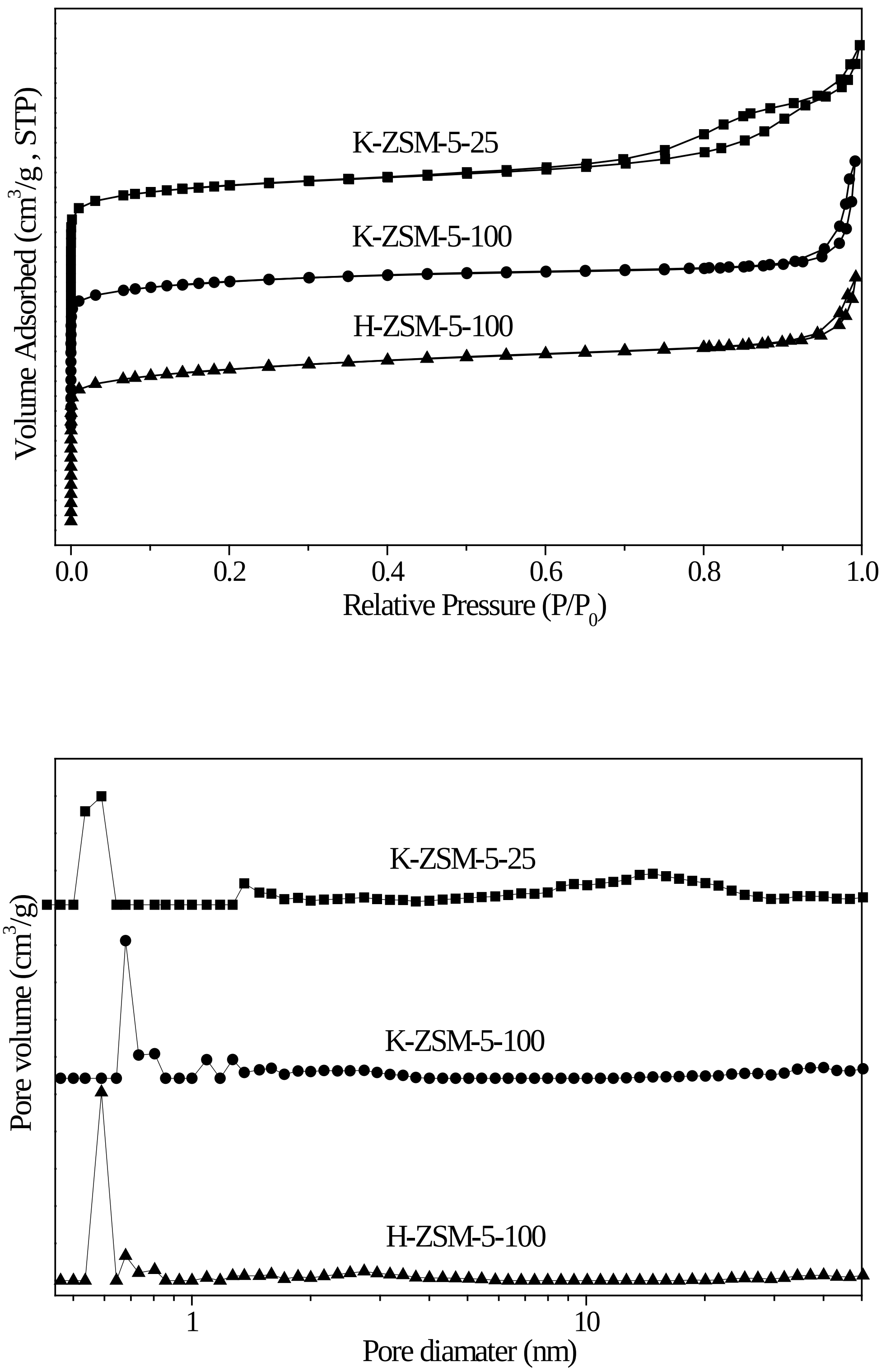

Figure S1. N_2 adsorption/desorption isotherms and pore size distribution of the samples.

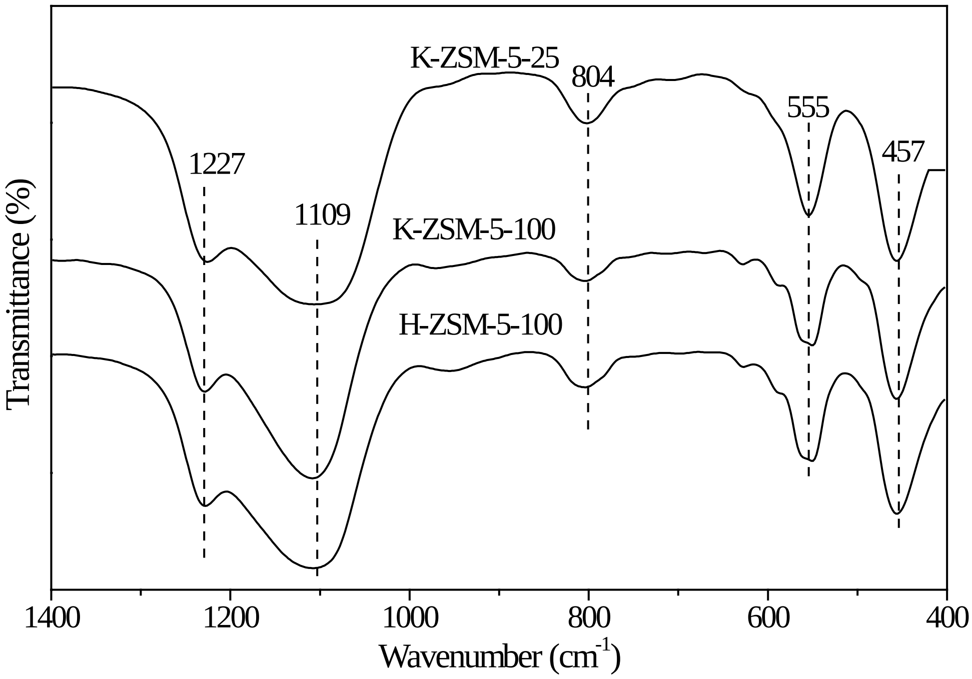

Figure S2. FTIR of the samples.

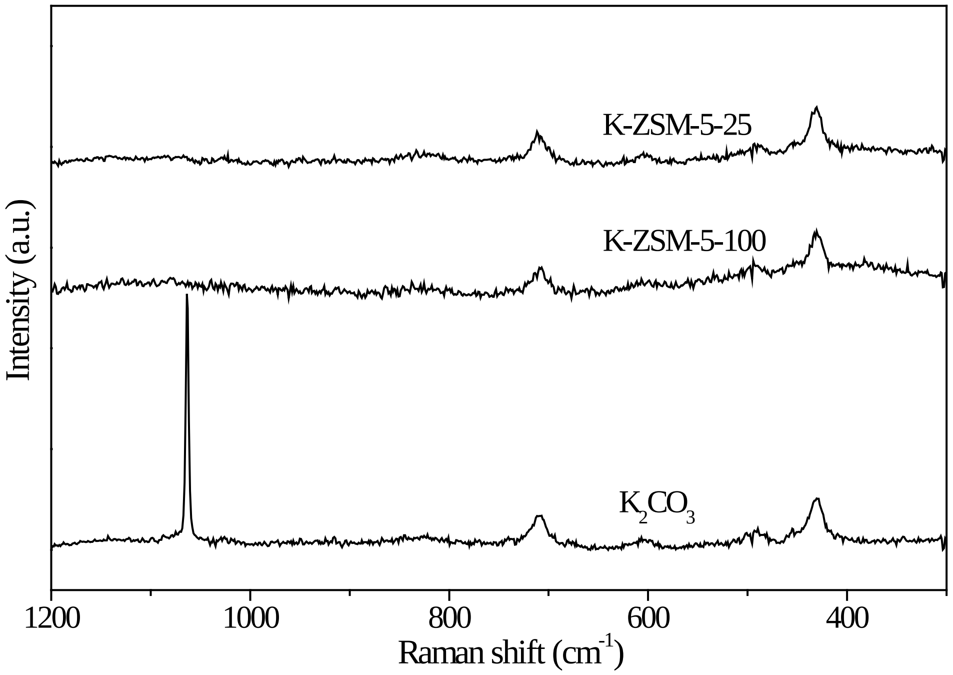

Figure S3. Raman spectra of K_2CO_3, K-ZSM-5-100 and K-ZSM-5-25.

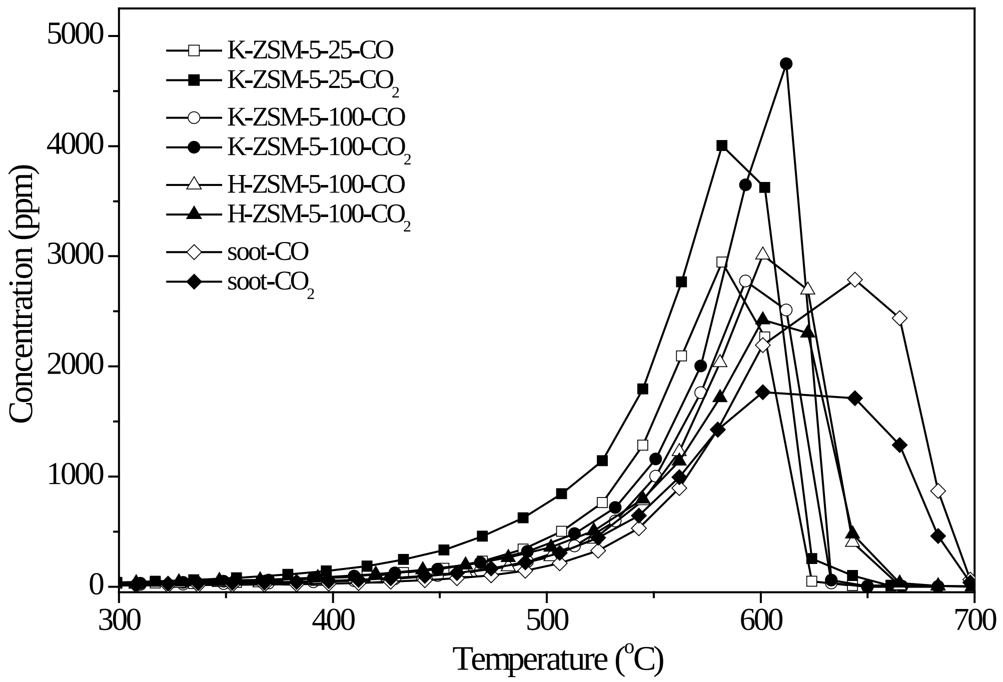

Figure S4. TPO curves of all samples.

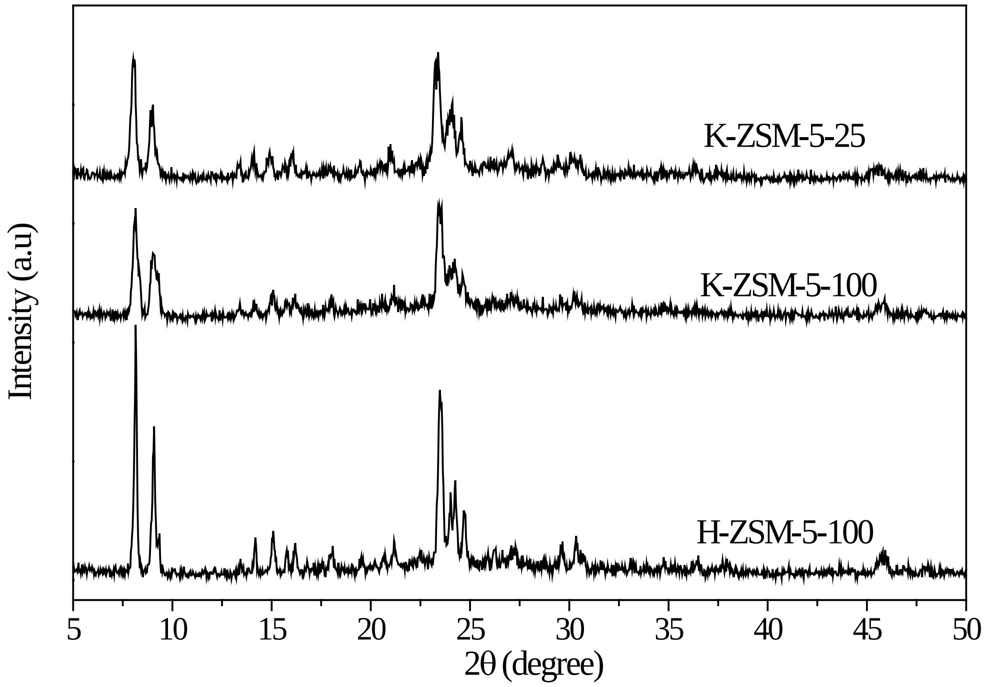

Figure S5. XRD patterns of the samples after TPO reactions.

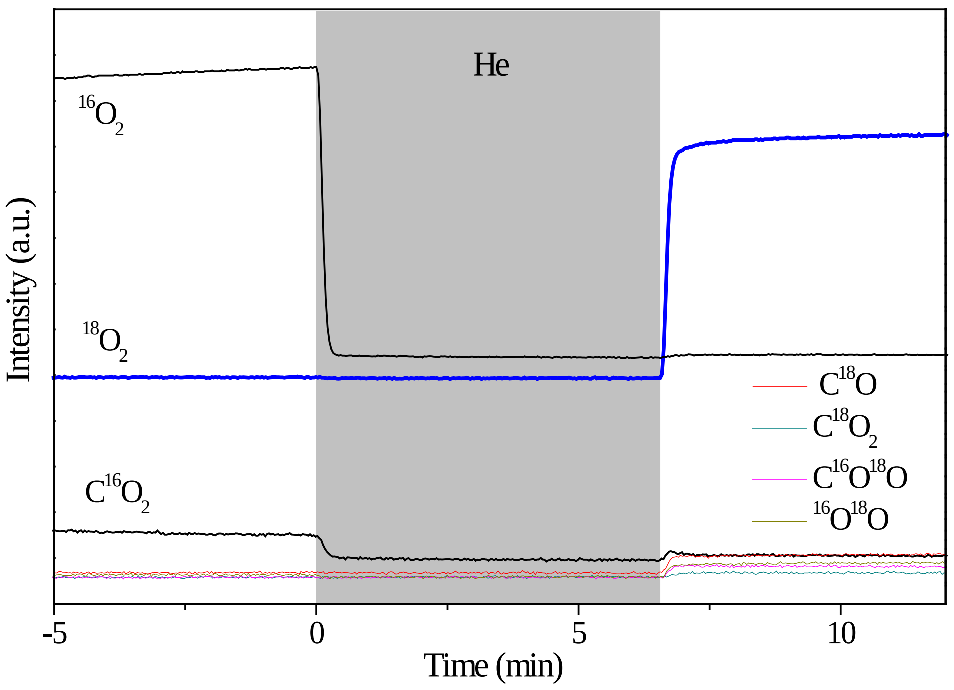

Figure S6. Isothermal reactions for soot combustion at 500 °C after 1%$^{16}O_2$ was switched to 1% $^{18}O_2$ in He on pure soot (diluted with SiO_2)
